# Exploring lysosomal biology: current approaches and methods

**DOI:** 10.52601/bpr.2023.230028

**Published:** 2024-04-30

**Authors:** Qiuyuan Yin, Chonglin Yang

**Affiliations:** 1 State Key Laboratory of Conservation and Utilization of Bio-resources in Yunnan and Center for Life Sciences, School of Life Sciences, Yunnan University, Kunming 650091, China; 2 Southwest United Graduate School, Kunming 650092, China

**Keywords:** Lysosome, Method, Cultured cell, *C. elegans*, Mice

## Abstract

Lysosomes are the degradation centers and signaling hubs in the cell. Lysosomes undergo adaptation to maintain cell homeostasis in response to a wide variety of cues. Dysfunction of lysosomes leads to aging and severe diseases including lysosomal storage diseases (LSDs), neurodegenerative disorders, and cancer. To understand the complexity of lysosome biology, many research approaches and tools have been developed to investigate lysosomal functions and regulatory mechanisms in diverse experimental systems. This review summarizes the current approaches and tools adopted for studying lysosomes, and aims to provide a methodological overview of lysosomal research and related fields.

## INTRODUCTION

Since its discovery by Christian de Duve in 1955, the lysosome has been proven to play central roles in cellular homeostasis (de Duve 2005). Bounded by a single bilayer phospholipid membrane, lysosomes are organelles that contain >50 hydrolytic enzymes, including proteases, nucleases, and lipases (Bagshaw *et al.* 2005; Muthukottiappan and Winter 2021). The lysosomal lumen is acidic with a pH of 4.6 to 5.2, which is necessary for the activity of hydrolytic enzymes. Lysosomes receive cargos generated by endocytosis, phagocytosis, or autophagy (Bright *et al.* 2016; Chen *et al.* 2010; Luzio *et al.* 2007). The degradation products are exported out of the lysosome and reused as building blocks to sustain cellular homeostasis. In addition, lysosomes interact with diverse intracellular organelles to participate in a wide range of cellular processes, including calcium homeostasis, lipid transfer, cholesterol homeostasis, exocytosis, and plasma membrane repair (Corrotte and Castro-Gomes 2019; Lloyd-Evans and Waller-Evans 2020; Meng *et al.* 2020; Saftig and Klumperman 2009; Tancini *et al.* 2020; Thelen and Zoncu 2017). Furthermore, lysosomes are now recognized to act as dynamic hubs for intracellular signal transduction (Perera and Zoncu 2016).

Dysfunction of lysosomes leads to lysosomal storage diseases (LSDs), which are characterized by the accumulation of undigested cargos within lysosomes (Platt *et al.* 2018). Lysosomes are also involved in neurodegenerative disorders (*e*.*g*., Alzheimer’s and Parkinson’s diseases), cancer progression and metastasis, as well as aging (Carmona-Gutierrez *et al.* 2016; Davidson and Vander Heiden 2017; Udayar *et al.* 2022). Thus, understanding the mechanisms of lysosome adaptation can provide invaluable information on developing therapeutic strategies for these diseases. Here we provide an overview of the major methodology used for deciphering lysosomal functions and homeostasis.

## STUDYING LYSOSOMES IN CULTURED CELLS

Many basic functions and properties of lysosomes are revealed in cultured mammalian cells. Antibodies against specific lysosomal proteins, dyes (especially pH-sensitive dyes), and genetically-encoded sensors are employed to reveal lysosomal identity, acidification, maturation, integrity, and numbers.

### Identifying lysosomes

**Figure 1:**
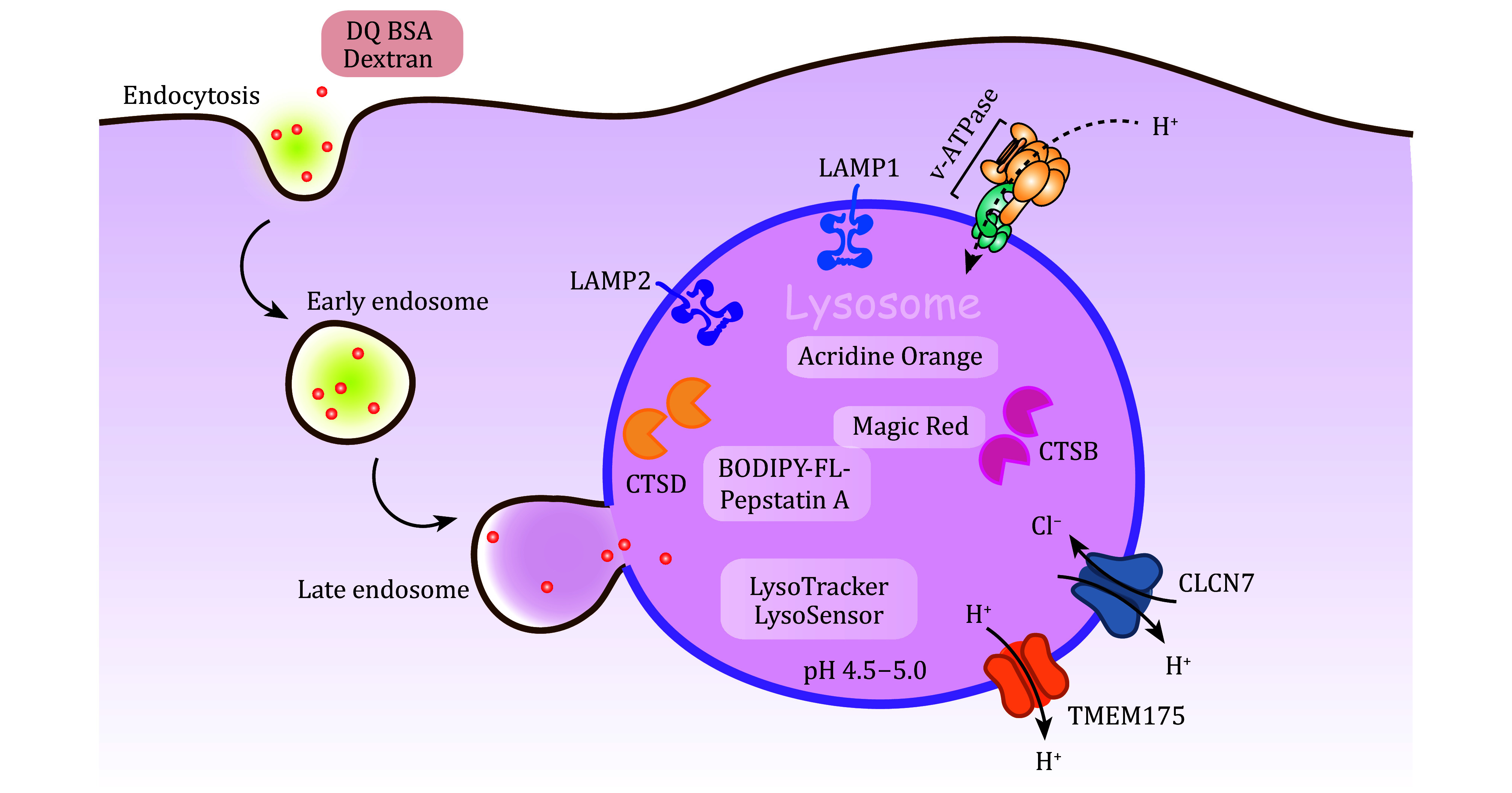
Commonly used fluorescent dyes and protein markers for lysosomes. The lysosomal integral membrane proteins LAMP1 and LAMP2, and the lysosomal acidic hydrolases CTSB (cathepsin B) and CTSD (cathepsin D), are commonly used for labeling lysosomes. The acidic lumen of lysosomes is maintained by the V-type proton ATPase (V-ATPase) and other ion transporters like CLCN7 and TMEM175. The fluorescent probes LysoTracker, LysoSensor and Acridine Orange are used in qualitative and quantitative analyses for lysosome identity and acidification. Magic Red and BODIPY-FL-Pepstatin A are fluorescent probes that indicate the maturation of lysosomes by targeting CTSB and CTSD, respectively. In addition, lysosomes can be marked by the extracellular cargos Dextran and DQ-BSA, which are internalized by endocytosis and delivered to lysosomes through the endosome-lysosome pathway

In fixed cells, immuno-staining with antibodies recognizing specific lysosomal proteins can identify lysosomes within a cell. The most frequently used proteins include the lysosomal integral membrane proteins LAMP1 and LAMP2, and the lysosomal acidic hydrolases cathepsin B (CTSB) and cathepsin D (CTSD) (Cheng *et al.* 2018; Saftig and Klumperman 2009; Schroder *et al.* 2010). Nevertheless, it is worth noting that sometimes it is not easy to distinguish between lysosomes and late endosomes as these two types of organelles share some common features (Luzio *et al.* 2007). Thus, additional immuno-staining with antibodies against late endosome proteins, such as Rab7 and LAMP3 (Hirst *et al.* 1998; Kobayashi *et al.* 2000; Liu *et al.* 2016), is recommended to distinguish lysosomes from late endosomes. In living cells, various pH-sensitive dyes, such as LysoTracker (Red or Green), LysoSensor (Red or Green), and Acridine Orange (Anderson and Orci 1988; Diwu *et al.* 1999; Eriksson *et al.* 2023; Lin *et al.* 2001), are used to label lysosomes, enabling real-time visualization and analysis of lysosomal morphology, dynamics, and pH variations. In addition, Dextran and DQ BSA are frequently used to mark lysosomes, as they can be internalized by endocytosis and delivered to lysosomes along the endosome-lysosome pathway. DQ BSA is a self-quenched albumin conjugate, which becomes fluorescent in the lysosomal lumen following cleavage by lysosome enzymes. This allows for quantitative measurement of lysosomal proteolytic activities. Dextrans are complex branched polysaccharides, which can be conjugated with various fluorescent dyes. Dextrans accumulate in lysosomes and are stable following internalization, making them ideal markers to monitor lysosomal dynamics (Fig. 1) (Frost *et al.* 2017; Lencer *et al.* 1990; Marwaha and Sharma 2017).

Live-cell imaging of lysosomes can be performed with ectopically expressed lysosomal proteins tagged with fluorescent proteins (Falcon-Perez *et al.* 2005; Farias *et al.* 2017; Xing *et al.* 2021). For long-term observations, establishing cell lines that stably express these fluorescent lysosomal markers will ensure extended observation times.

### Investigating lysosome acidification

The acidic lumen of lysosomes is cooperatively regulated by the V-type proton ATPase (V-ATPase) and other ion channels like CLCN7 and TMEM175 (Hu *et al.* 2022; Mindell 2012). The acidification of the lysosomal lumen guarantees the processing and activation of lysosomal hydrolytic enzymes.

The fluorescent probes LysoTracker and LysoSensor have been used for measuring lysosomal pH. Among them, LysoTracker probes, such as DND-99 and DND-26, offer a straightforward method for qualitative measurement of lysosome acidification (Anderson and Orci 1988). LysoSensor probes, in comparison, are suitable for more accurate analysis of lysosomal pH (Diwu *et al.* 1999). Notably, the LysoSensor yellow/blue DND-160 probe allows for lysosomal pH measurement by emitting yellow and blue fluorescence in acidic and neutral environments, respectively (DePedro and Urayama 2009). LysoSensor Blue DND-167 and LysoSensor Green DND-189 have a p*K*a of ~5.1, and thus are almost non-fluorescent unless in the acidic compartments. Additionally, pH-sensitive fluorophores, such as pHrodo (p*K*a ~6.8), fluorescein isothiocyanate (FITC) (p*K*a ~6.4), and Oregon Green (p*K*a ~4.8), have been developed to evaluate lysosomal pH (Oben and Foreman 1988). Conjugating these fluorophores with a fluid-phase dextran marker enables detailed and prolonged imaging of acidic lysosomes.

**Figure 2:**
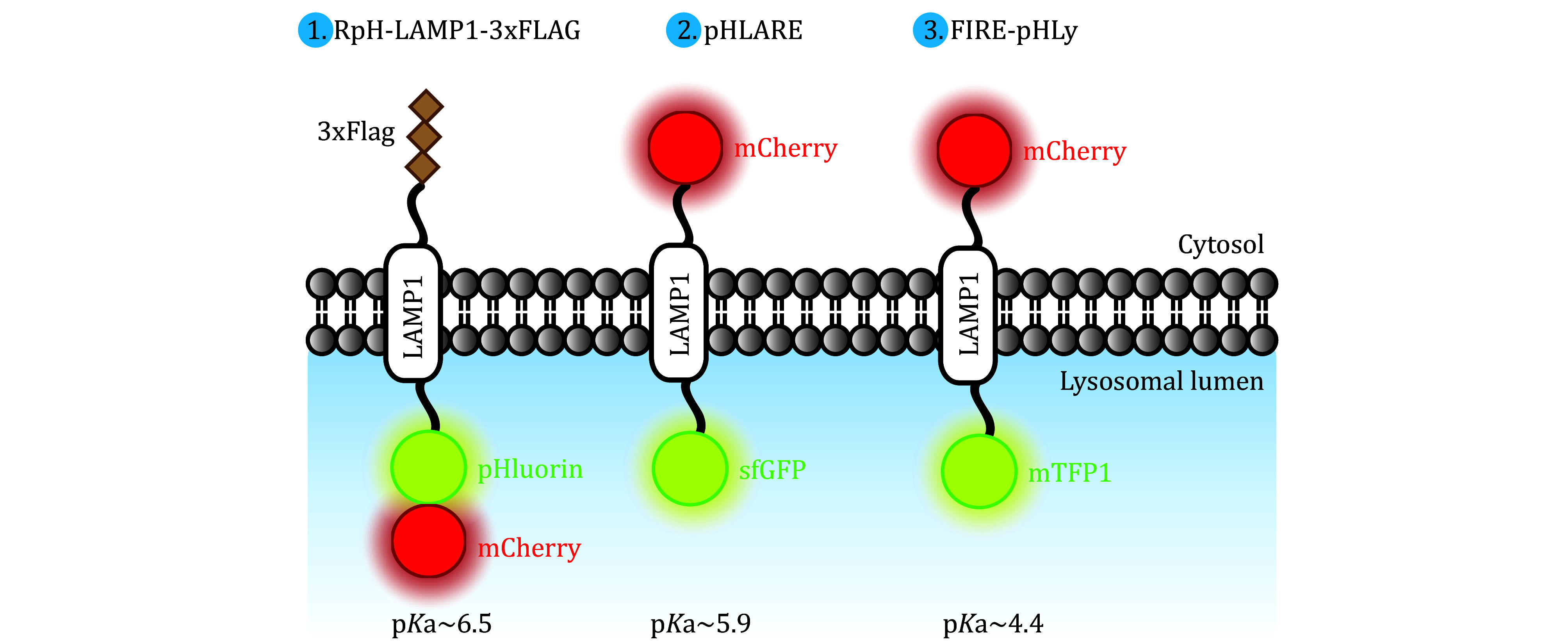
Genetically-encoded probes for measurement of lysosomal pH. Genetically-encoded probes for lysosomal pH assessment are developed by combining pH-sensitive GFP variants and pH-insensitive mCherry, and tagging them onto LAMP1. GFP signals are observed in endosomes of higher pH but are quenched in the acidic lysosomes, while mCherry fluorescence is detectable in both compartments. The schematic diagram shows three lysosomal pH probes with different p*K*a values: (1) RpH-LAMP1-3xFLAG (p*K*a ~6.5); (2) pHLARE (p*K*a ~5.9); (3) FIRE-pHLy (p*K*a ~4.4)

Genetically-encoded probes, which typically combine pH-sensitive GFP variants and pH-insensitive mCherry and are tagged onto target proteins, are also applicable to lysosomal pH assessment. GFP signals are observed in endosomes of higher pH but are quenched in the acidic lysosomes. In contrast, mCherry fluorescence remains detectable in both compartments. Examples of such probes include (1) a fusion of pHluorin and mCherry attached to the luminal domain of LAMP1 (RpH-LAMP1-3xFLAG) (Ponsford *et al.* 2021); (2) LAMP1 tagged with superfolder GFP at the luminal domain and mCherry at the cytosolic domain (pHLARE) (Webb *et al.* 2021); (3) LAMP1 tagged with monomeric teal fluorescent protein 1 (mTFP1) at the luminal domain and mCherry at the cytosolic domain (FIRE-pHLy) (Chin *et al.* 2021); (4) mRFP-GFP tandem fluorescent-tagged LC3B (tfLC3), which exhibits only the mRFP signal in lysosomes (Kimura *et al.* 2007). These ratiometric fluorescent probes provide reliable methods for long-term monitoring of lysosomal acidification (Fig. 2).

Lysosomal inhibitors are important tools for dissecting lysosomal function and homeostasis. Bafilomycin A1 (BFA1) and Concanamycin A inhibit V-ATPase, preventing lysosomal acidification (Huss *et al.* 2002; Yoshimori *et al.* 1991). Chloroquine (CQ), hydroxychloroquine (HCQ), and ammonium chloride (NH_4_Cl) are weak bases that accumulate in lysosomes and raise the pH to inhibit lysosomal enzymes (Hart and Young 1991; Homewood *et al.* 1972; Titus 1989). Other inhibitors, such as Leupeptin and Pepstatin A, impact lysosomal function by inhibiting protease activities (Kuroda *et al.* 1994).

### Understanding lysosome maturation

Lysosome maturation can be reflected by the activities of hydrolytic enzymes, such as cathepsins (Turk *et al.* 2012). Magic Red and BODIPY-FL-Pepstatin A are fluorescent probes and substrates of lysosomal hydrolases. Magic Red emits a red fluorescent signal upon cleavage by active cathepsin B in mature lysosomes (Boonacker and van Noorden 2001; van Noorden *et al.* 1997). BODIPY-FL-Pepstatin A is a synthetic fluorescent dye that selectively binds to cathepsin D at pH 4.5 (Chen *et al.* 2000) (Fig. 1).

Biochemical assays, including ELISA and western blotting, are used to examine the levels of cathepsins and their activities. Cathepsins are initially produced as inactive pro-cathepsins, which are converted into the mature forms via proteolytic cleavage (Yadati *et al.* 2020). For instance, the cleavage of pro-cathepsin D first generates an intermediate single-chain form. Following further processing and assembly, the cathepsin D intermediate is converted into a double-chain, mature form of cathepsin D (Gieselmann *et al.* 1985). Commercially available ELISA kits can be applied to various sample types to detect total cathepsin activities.

Overall, the status of lysosomal maturation is judged by multiple assays as summarized above.

### Exploring lysosome integrity

Lysosomal integrity is a prerequisite for lysosomal function. To examine lysosomal integrity, the following approaches can be utilized. (1) Lysosomal membrane permeabilization (LMP) assays. This method assesses lysosome damage by detecting the permeability of lysosomal membranes, the resulting increase in lysosomal pH, and the release of lysosomal enzymes. pH-sensitive dyes or genetically-encoded pH probes can be used to assess the pH of intact and damaged lysosomes. In addition, the activities of cathepsins can be measured by using specific substrates that generate fluorescent or colorimetrically detectable products (Wang *et al.* 2018). (2) Lysosome labeling by galectin-3. Galectin-3 is a cytosolic protein that selectively binds to β-galactoside residues exposed on the inner surface of damaged lysosomal membranes. For example, L-leucyl-L-leucine methyl ester (LLOMe) is a lysosomotropic agent which permeabilizes the lysosomal membrane. Following LLOMe treatment of cells, EGFP-Galectin-3 accumulates in damaged lysosomes and is visualized with fluorescence microscopy (Aits *et al.* 2015; Jia *et al.* 2020). (3) Transmission electron microscopy (TEM). TEM provides ultrastructural details of intact and damaged lysosomes.

### Investigating lysosome biogenesis

Lysosomes increase their numbers to meet cellular demands for degradation and in response to a wide variety of signals. TFEB/TFE3 are two major transcription factors that promote lysosome biogenesis. Following a range of different signaling events, TFEB/TFE3 translocate into the nucleus to activate the transcription of lysosomal genes (Cinque *et al.* 2020; Khaminets *et al.* 2015; Maejima *et al.* 2013; Pickles *et al.* 2018; Roczniak-Ferguson *et al.* 2012; Sardiello *et al.* 2009).

To evaluate lysosome biogenesis, the following points can be considered. (1) Lysosome numbers. Typically, lysosomes are stained with fluorescent dyes such as LysoTracker, LysoSensor, MagicRed, BODIPY-FL-Pepstatin A, and dextran beads. Thus, lysosomes in living cells can be scored under fluorescence microscopy or quantified with flow cytometry. In fixed cells, lysosomes can be quantified by immunostaining of lysosomal proteins, such as LAMP1, LAMP2, and LIMPII (also called LIMP-2 or SCARB1) (Li *et al.* 2016b; Yin *et al.* 2020). Changes in lysosomal protein levels (*e*.*g*., LAMP1, LAMP2, cathepsin D), detected by western blotting, may also reflect changes in lysosome numbers. Nevertheless, it is suggested that additional evidence be taken into account as the increase in lysosomal protein levels might suggest alterations in lysosome size or protein contents. (2) TFEB/TFE3 activation. Under normal conditions, TFEB/TFE3 localize in the cytoplasm and are in the phosphorylated (inactive) state. Under certain circumstances, *i*.*e*., starvation, TFEB/TFE3 are activated by dephosphorylation and they translocate into the nucleus where they activate lysosomal and autophagy gene expression (Puertollano *et al.* 2018; Raben and Puertollano 2016). The phosphorylation status of TFEB/TFE3 can be investigated by electrophoretic mobility shift assays. In addition, TFEB/TFE3 phosphorylation can be determined with antibodies against specific phosphorylation sites (*i*.*e*., TFEB phospho-Ser142 and phospho-Ser211 antibodies). Translocation of TFEB/TFE3 is easily assessed with immunostaining of the endogenous proteins or ectopically expressed TFEB/TFE3 tagged with fluorescent proteins. (3) TFEB/TFE3 transcriptional activity. Once in the nucleus, TFEB/TFE3 bind to the CLEAR (Coordinated Lysosomal Expression and Regulation) motif of the target genes (Settembre *et al.* 2011). qPCR is commonly used to examine if TFEB/TFE3 target genes are upregulated. Potential TFEB/TFE3 binding sites within a gene promoter region can be predicted by using the “matchPWM” or JASPAR databases (Castro-Mondragon *et al.* 2022). Experimentally, a reporter plasmid with luciferase or GFP driven by the predicted promoter can be co-expressed with TFEB/TFE3 expression vectors to analyze the transcriptional activity. Chromatin immunoprecipitation (ChIP) and qPCR can further validate TFEB-promoter binding specificity and transcriptional activities.

## *CAENORHABDITIS ELEGANS* AS A MODEL TO STUDY LYSOSOMES

The nematode *C. elegans*, with its short life cycle, well-characterized genome, and powerful genetic tools, provides an excellent and unique system for genetic and cell biological dissection of lysosomal homeostasis and functions (Gan *et al.* 2019; Li *et al.* 2016a; Liu *et al.* 2012, 2016; Xu *et al.* 2014; Yang and Wang 2017, 2021). Moreover, the requirement for lysosomes in animal development and aging is reflected in a straightforward way in *C. elegans*.

### Visualizing lysosomes in *C. elegans*

In *C. elegans*, lysosomes can be labeled by transgenic expression of lysosomal proteins tagged with fluorescent proteins (*e*.*g*., GFP, mCherry, etc.). Lysosomal proteins, such as NUC-1 (DNase) and SCAV-3 (LIMPII), are commonly used (Guo *et al.* 2010; Li *et al.* 2016a). Additional lysosomal proteins include LAAT-1 (Lysosomal amino acid transporter 1), CPL-1 (cathepsin L), CUP-5 (TRMPL1), and CTNS-1 (cystinosin, lysosomal cystine transporter) (Liu *et al.* 2012; Xu *et al.* 2014). In wild-type worms, lysosomes labeled with LAAT-1::GFP and NUC-1::CHERRY exhibit vesicular and tubular structures over distinct developmental stages and tissues (Li *et al.* 2016a; Liu *et al.* 2012). Lysosomal fluorescent dyes and probes, such as LysoTracker and LysoSensor, are also applicable in *C. elegans* studies (Li *et al.* 2016a; Miao *et al.* 2020; Sun *et al.* 2020).

TEM and high-voltage electron microscopy (HVEM) reveal detailed lysosomal ultrastructures in *C. elegans*. With electron microscopy, several lysosome types have been identified in both wild-type and lysosomal gene mutants, providing invaluable insights into gene functions (Li *et al.* 2016a; Miao *et al.* 2020; Sun *et al.* 2020).

### Tracking lysosomal maturation and acidification in *C. elegans*

**Figure 3:**
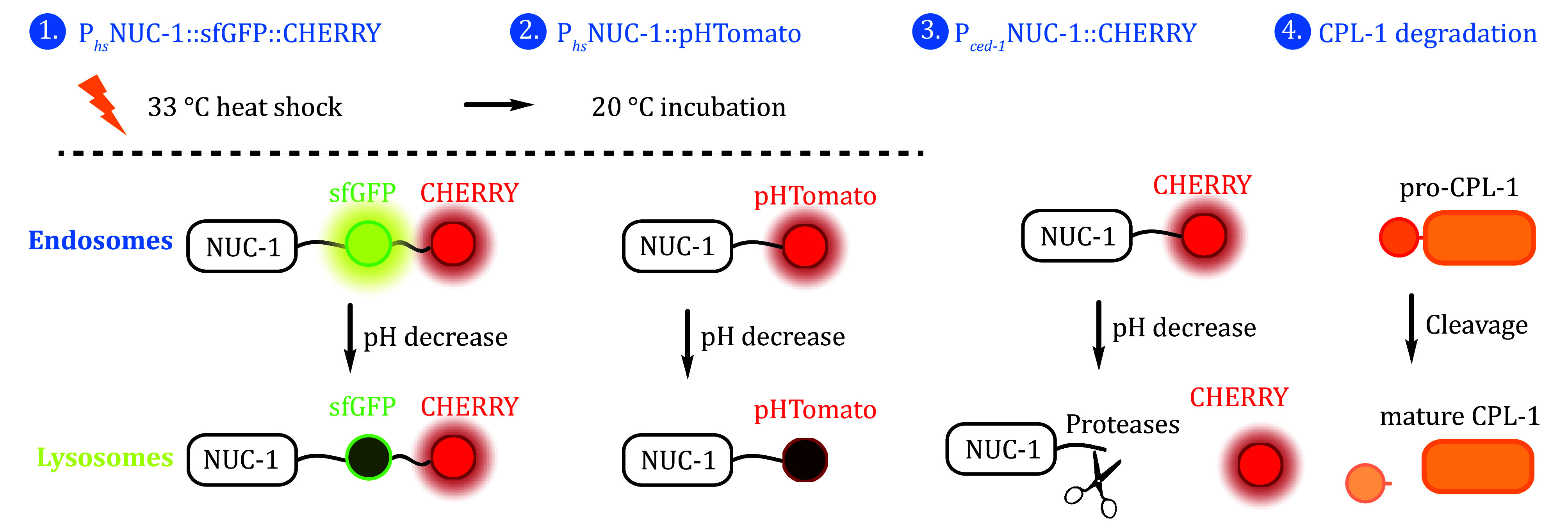
*In vivo* markers used to track lysosomal maturation and acidification in *C. elegans*. (1) P_*hs*_NUC-1::sfGFP::CHERRY. Lysosomal maturation can be monitored using transgenic arrays expressing the lysosomal hydrolase NUC-1 tandemly tagged with GFP and CHERRY, driven by the heat-shock (hs) promoter. (2) P_*hs*_NUC-1::pHTomato. Lysosomal acidification can be assessed by fusing NUC-1 with the pH-sensitive fluorescent protein pHTomato. pHTomato signals increase when the pH rises. (3) P_*ced-1*_NUC-1::CHERRY. Lysosomal degradation activity is evaluated by examining the cleavage of the NUC-1::CHERRY fusion protein. Marker expression is driven by the *ced-1* promoter, which is active in engulfing cells. (4) Cathepsin L (CPL-1). Lysosomal degradation activity is also assessed by monitoring the processing of CPL-1. In degradation-competent mature lysosomes, the pro-domain of pro-CPL-1 is cleaved to yield mature CPL-1

Several assays have been developed to investigate lysosomal maturation, acidification, and cleavage activity. Lysosomal maturation can be monitored using transgenic arrays expressing the lysosomal hydrolase NUC-1 tandemly tagged with GFP and CHERRY, driven by the heat-shock (hs) promoter (P_*hs*_NUC-1::sfGFP::CHERRY). After heat shock triggers NUC-1::sfGFP::CHERRY expression, the tandem fusion protein is delivered to lysosomes along the endosome-lysosome pathway. Because GFP signals are quenched in acidic lysosomes, the acidification and maturation of lysosomes are inferred from the GFP-negative and CHERRY-positive structures (Miao *et al.* 2020). Lysosomal acidification can also be assessed by using the pH-sensitive fluorescent protein pHTomato fused with NUC-1 (P_*hs*_NUC-1::pHTomato). The fluorescence of pHTomato, which has a p*K*a of approximately 7.8, increases when the pH rises (Wang *et al.* 2019). Lastly, lysosomal degradation activity is evaluated by examining the cleavage of the NUC-1::CHERRY fusion protein and the processing of Cathepsin L (CPL-1). Lysosomal proteases cleave CHERRY from the NUC-1::CHERRY fusion protein, and the resultant CHERRY protein levels can be determined by western blot. Cathepsin L (CPL-1) is initially produced as an inactive precursor, which undergoes proteolytic cleavage to remove the pro-domain, thus yielding the mature form. Lysosomal degradation activity can be assessed by analyzing the processing of endogenous CPL-1 with western blotting assays (Fig. 3) (Miao *et al.* 2020; Xu *et al.* 2014).

### Studying lysosomal integrity in *C. elegans*

Fluorescent markers and dyes, such as LysoTracker and LysoSensor, as well as genetically-encoded probes (*i*.*e*., NUC-1::sfGFP::CHERRY and NUC-1::pHTomato), are frequently used to investigate damaged lysosomes. When NUC-1::sfGFP::CHERRY is used, compartments that exhibit yellow fluorescence (both GFP- and CHERRY-positive) are thought to be abnormal lysosomes (Miao *et al.* 2020; Wang *et al.* 2019). TEM provides visual details of lysosomal damage. For example, damaged lysosomes may exhibit swollen, ruptured, and irregular structures (Li *et al.* 2016a). Lysosomal membrane breakage leads to the exposure of luminal glycoproteins. Thus, transgenic expression of GFP-tagged galectin proteins, GFP::Gal3 and GFP::Gal9, enables the visualization of lysosomal damage. In wild-type animals without lysosomal damage, GFP::Gal3 and GFP::Gal9 are distributed in the cytoplasm. However, in animals carrying the *scav-3*(*qx193*) mutation, which destabilizes lysosomal membranes, the galectin reporters accumulate into puncta in the damaged lysosomes (Li *et al.* 2016a).

### Monitoring lysosomal dynamics in *C. elegans*

The use of genetic fluorescent reporters to label lysosomes in *C. elegans* enables the tracking of lysosome dynamics in living animals. With time-lapse imaging, it is feasible to quantitatively analyze lysosomal fusion, fission, and movement in distinct cell types, developmental and aging stages, and stress conditions. The Pearson’s correlation coefficient quantifies lysosomal movement by measuring the colocalization between two consecutive time-lapse frames taken 30–60 s apart. It was found that lysosomes are more dynamic at larval stages, with notable increases during the molting stage. Lysosomal dynamics tends to decrease with aging (Miao *et al.* 2020; Sun *et al.* 2020).

## STUDYING LYSOSOMES IN MICE

Histological and *in vivo* imaging studies in mice offer a comprehensive understanding of lysosomal function at the organism level.

**Figure 4:**
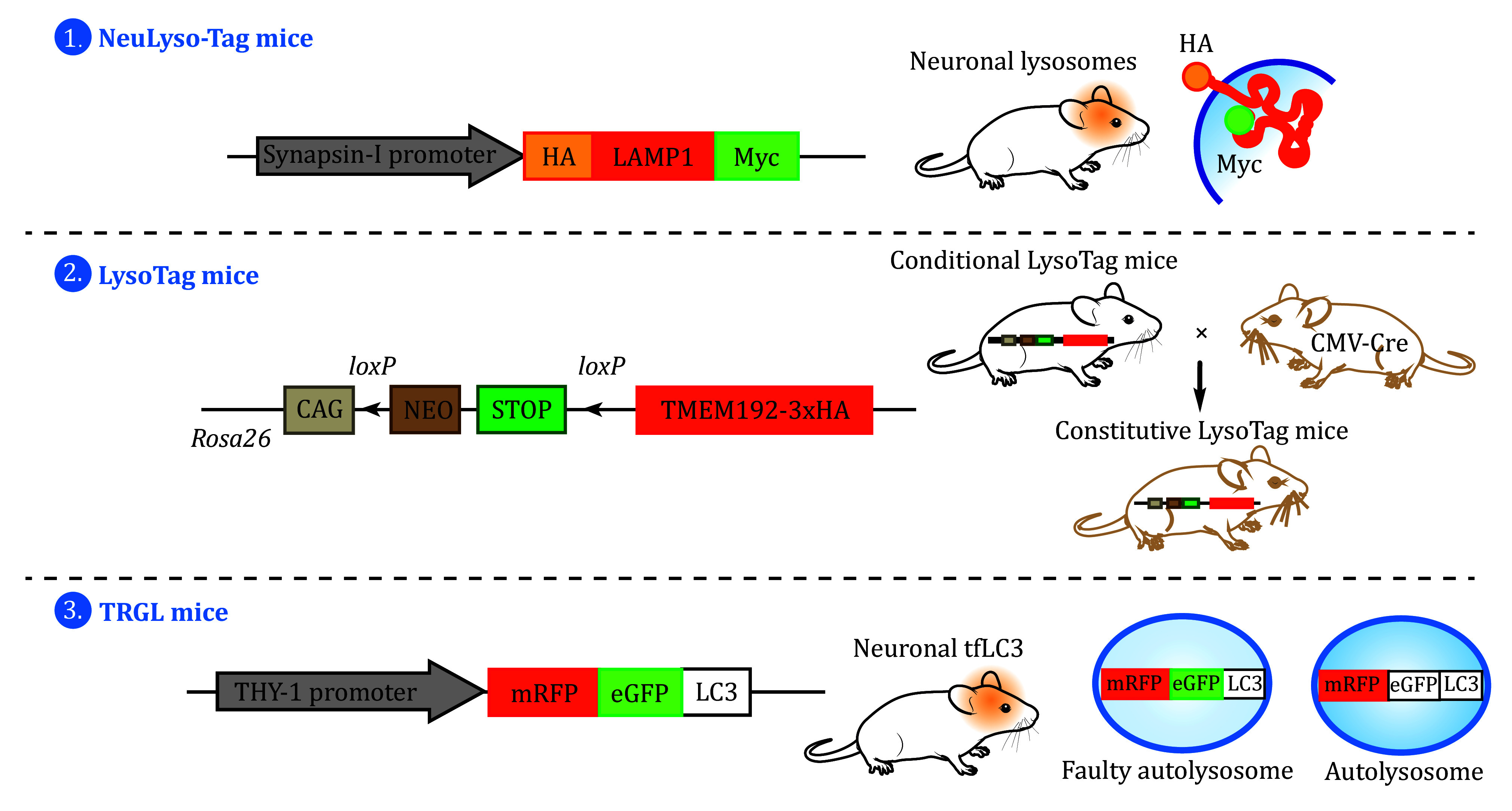
Schematic representation of transgenic mice expressing autophagic or lysosomal proteins for the study of lysosomes. (1) Transgenic “NeuLyso-Tag” mice express neuron-specific ^HA^LAMP1^Myc^ driven by the synapsin-I promoter. (2) Conditional “LysoTag” transgenic mice carry a TMEM192-3 × HA-LSL cassette. Constitutive “LysoTag” mice are generated when the conditional mice are bred with those carrying the CMV-Cre transgene. (3) TRGL mice express neuron-specific mRFP-eGFP-LC3 under the control of the Thy1.1 promoter

Several labs have developed transgenic mice expressing autophagic or lysosomal proteins, facilitating the study of lysosomes in mammals. Transgenic mice have been generated that express neuron-specific ^HA^LAMP1^Myc^ (“NeuLyso-Tag”), in which LAMP1 is tagged with 2xHA at the luminal domain and 2xMyc at the cytoplasmic tail (Xie *et al.* 2022). The expression of ^HA^LAMP1^Myc^ is driven by the neuron-specific synapsin-I promoter, allowing for the targeted affinity-isolation of neuronal late endosomes/lysosomes from mouse brains. In addition, a transgenic “LysoTag” mouse line was developed, which integrated a lysosomal TMEM192-3 × HA fusion protein into the Rosa26 locus downstream of a lox-stop-lox (LSL) cassette. Constitutive “LysoTag” mice are generated when the mice carrying the lysosomal TMEM192-3 × HA-LSL cassette are bred with those carrying the CMV-Cre transgene, such that TMEM192-3 × HA is expressed across various tissues, allowing for immunostaining or isolation of lysosomes by using anti-HA antibodies (Laqtom *et al.* 2022). To study autophagy and lysosomal functions *in vivo*, transgenic TRGL mice were generated, which express neuron-specific mRFP-eGFP-LC3 (Thy-1 mRFP-eGFP-LC3), in which tfLC3 is integrated into the Thy1.1 expression cassette (Lee *et al.* 2022; Mizushima *et al.* 2004). With the TRGL mice, it was found that neuronal autolysosome acidification diminishes before the onset of extracellular amyloid deposition (Fig. 4).

Mouse models with targeted gene knockout or overexpression are important for exploring lysosomal functions and related diseases. For instance, mouse models have been developed that mimic LSDs such as Gaucher (*Gba1*), Pompe (*Gaa*), and Niemann-Pick (*Npc1/2*) diseases (Elrick *et al.* 2010; Liou *et al.* 2019; Raben *et al.* 1998). In addition, the transcription factor TFEB has been studied by using tissue-specific knockout mice since a complete TFEB knockout is lethal (Steingrimsson *et al.* 1998). TFEB/TFE3 can also be overexpressed in mice through helper-dependent adenoviruses (HDAd) or adeno-associated viruses (AAV) in specific tissues (Doronzo *et al.* 2019; Mansueto *et al.* 2017; Settembre *et al.* 2013). With these mouse models, it was found that TFEB/TFE3 play essential roles in metabolism, vascular development, and organ growth and regeneration.

## CONCLUSION AND PERSPECTIVE

The application of many different lysosome-specific tools and assays in cultured cells, *C. elegans* and mice, as summarized above, has greatly facilitated the understanding of lysosome function and regulation under physiological and pathological conditions. While the well-established tools will continue to contribute to the study of lysosomal biology, it is necessary to develop novel assays for a better understanding of lysosomal functions in diverse physiological and pathological conditions. This is particularly important for exploring lysosomal functions and dynamics in animal embryogenesis, development and aging, as well as in stem cell self-renewal and differentiation (Yang and Wang 2021).

## Conflict of interest

Qiuyuan Yin and Chonglin Yang declare that they have no conflict of interest.
